# Supplementary material for: Illuminating the dark space of neutral glycosphingolipidome by selective enrichment and profiling at multi-structural levels
Source: Nat Commun. 2024 Jul 4;15:5627. doi: 10.1038/s41467-024-50014-8 (PMC11224418; doi:10.1038/s41467-024-50014-8)
Supplement: Supplementary file 1 — Supplementary Information [file 41467_2024_50014_MOESM1_ESM.pdf]

Supplementary Information for

Illuminating the dark space of neutral glycosphingolipidome by selective enrichment and profiling at multi-structural levels

Wang et al.

Contents:

Supplementary Figures 1-22

Supplementary Notes 1-8

Supplementary Tables 1-3

Supplementary Methods

Supplementary References

Supplementary Figures, Supplementary Notes:

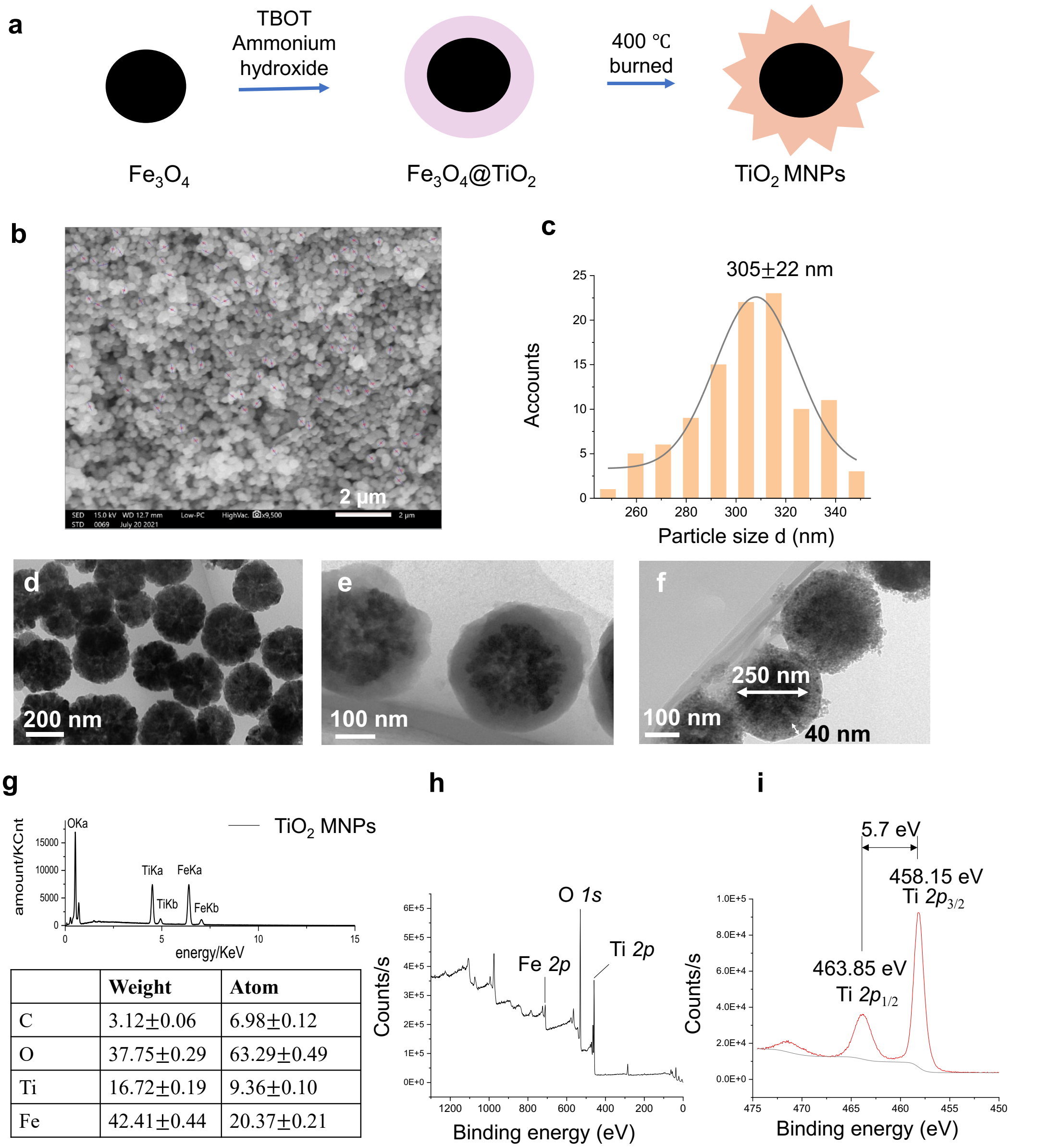

	Weight	Atom
C	3.12±0.06	6.98±0.12
O	37.75±0.29	63.29±0.49
Ti	16.72±0.19	9.36±0.10
Fe	42.41±0.44	20.37±0.21

Supplementary Figure 1. Synthesis and characterization of TiO_2 magnetic nanoparticles (MNPs). a. Decoration of a mesoporous TiO_2 layer on the ferrite core. b. Scanning Electron Microscope (SEM) image of TiO_2 MNPs. c. Size distribution of 105 TiO_2 MNPs, analyzed from the image in Figure S2e. Transmission electron microscopy (TEM) images of d. Fe_3O_4 core, e. Fe_3O_4@TiO_2, and f. TiO_2 magnetic nanoparticles after calcination. g. Energy-dispersive X-ray spectrometry (EDX) analysis of TiO_2 MNPs. h. Full scan X-ray photoelectron (XPS) spectrum of TiO_2 MNPs and (i) Ti *2p* XPS spectrum of TiO_2 MNPs.

Supplementary Note 1: The two peaks centered at 463.85 and 458.15 eV in Fig 1i are well corresponded to Ti $2p_{1/2}$ and Ti $2p_{3/2}$ binding energies. And the splitting energies between them is 5.7 eV, indicating a normal state of Ti^{4+} in the TiO_2 MNPs.

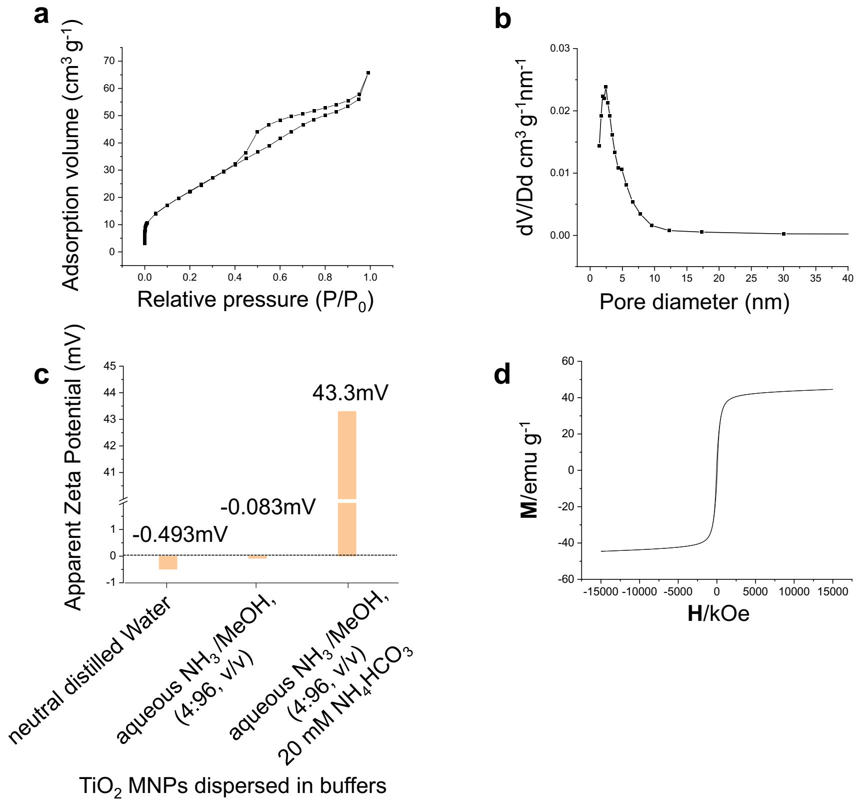

Supplementary Figure 2. Characterization of TiO_2 MNPs. a. N_2 sorption–desorption isotherms. b. Pore size distribution analysis obtained using the Barrett–Joyner–Halenda (BJH) method. c. Zeta potentials of TiO_2 MNPs in three buffers: neutral distilled water, aqueous NH_3/MeOH (4:96, v/v) with a solution pH of 11.33, aqueous NH_3/MeOH (4:96, v/v) containing 20 mM NH_4HCO_3 with a solution pH of 10.18. d. Magnetic hysteresis curves of TiO_2 MNPs.

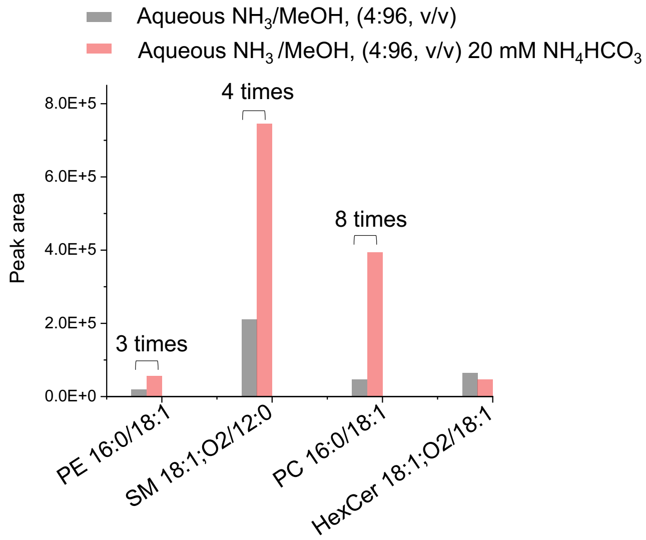

Supplementary Figure 3. Optimization of washing buffer. The mixture of standard lipids were prepared with a combination of PE 16:0/18:1, PE 16:0/18:1, SM 18:1;O2/12:0, and HexCer 18:1;O2/18:1, with a ratio of 5:5:5:1. The bars illustrate the LC peak area of $[M + H]^+$ ions of each

lipid in the washing buffer with or without 20 mM NH_4HCO_3. The increased abundances of phospholipids in different washing buffer solutions are indicated above the bars. Source data are provided in a Source Data file.

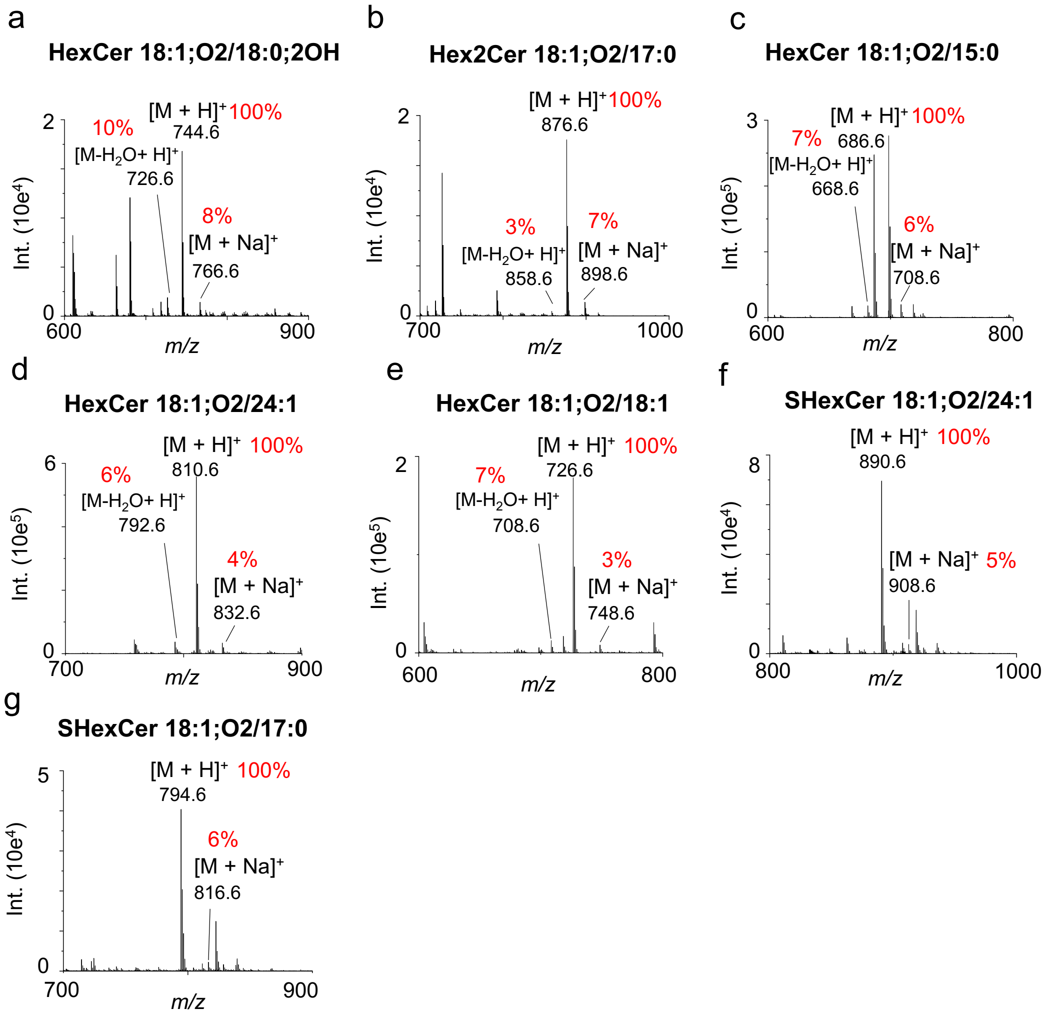

Supplementary Figure 4. MS^1 spectra of standard GSLs after selective enrichment. The relative abundance of $[M - H_2O + H]^+$, $[M + H]^+$, and $[M + Na]^+$ peaks of 1.25 μM of (a) HexCer 18:1;O2/18:0;2OH, (b) Hex2Cer 18:1;O2/17:0, (c) HexCer 18:1;O2/15:0, (d) HexCer 18:1;O2/24:1, and (e) HexCer 18:1;O2/18:1. The relative abundance of $[M + H]^+$, and $[M + Na]^+$ peaks of (f) SHexCer 18:1;O2/24:1 and (g) SHexCer 18:1;O2/17:0.

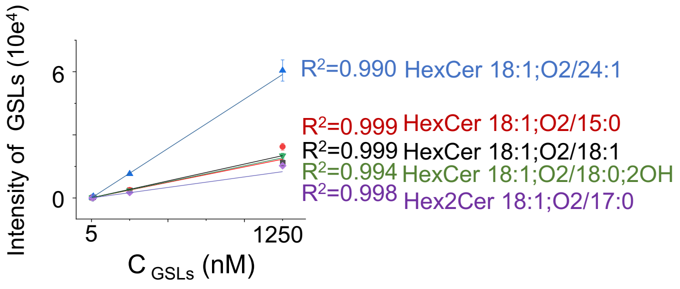

Supplementary Figure 5. MS responses of the enriched GSLs as a function of different initial concentrations (5, 12.5, 250, and 1250 nM). Error bars illustrate ± standard deviations (SD) from 3

technical replicates. Source data are provided in the Source Data file.

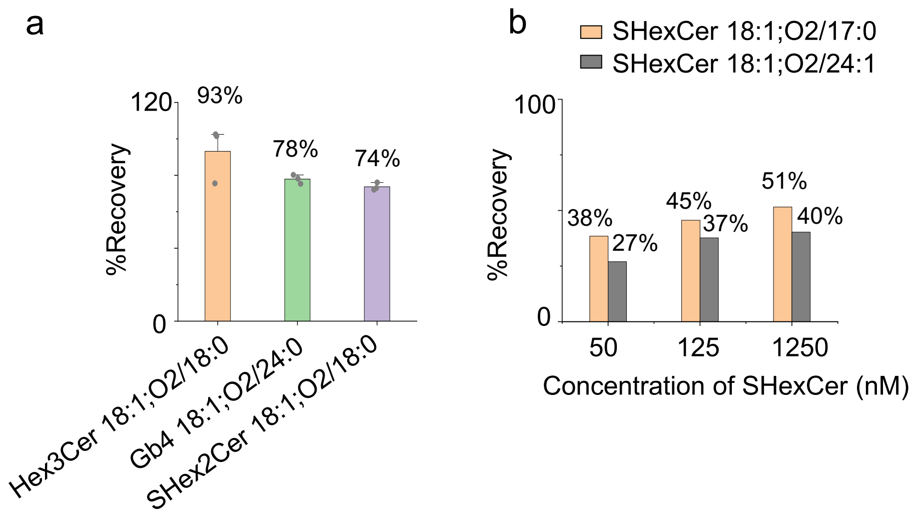

Supplementary Figure 6. %Recovery of five GSL standards (Hex3Cer 18:1;O2/18:0, Gb4 18:1;O2/24:0, SHex2Cer 18:1;O2/18:0, SHexCer 18:1;O2/17:0, SHexCer 18:1;O2/24:1) from standard lipid mixtures. a. %Recovery of lipid standards: Hex3Cer 18:1;O2/18:0, Gb4 18:1;O2/24:0, and SHex2Cer 18:1;O2/18:0 in a lipid mixture. The mixture contains 30 μM of phosphatidylcholine (PC), phosphatidylethanolamine (PE), and sphingomyelin (SM), respectively, along with initial concentrations of Hex3Cer, Gb4, and SHexCer at 1,000 nM. Error bars illustrate ± standard deviations (SD) from 3 technical replicates. b. %Recovery of lipid standards: SHexCer 18:1;O2/17:0, SHexCer 18:1;O2/24:1) in lipid mixtures. The mixtures are prepared with 20 to 1,000 times higher amounts of phosphatidylcholine (PC), phosphatidylethanolamine (PE), and sphingomyelin (SM), along with SHexCer initial concentrations of 50 nM, 125 nM, and 1,250 nM, respectively. The bars illustrate the mean values from 2 technical replicates. Source data is provided in a Source Data file.

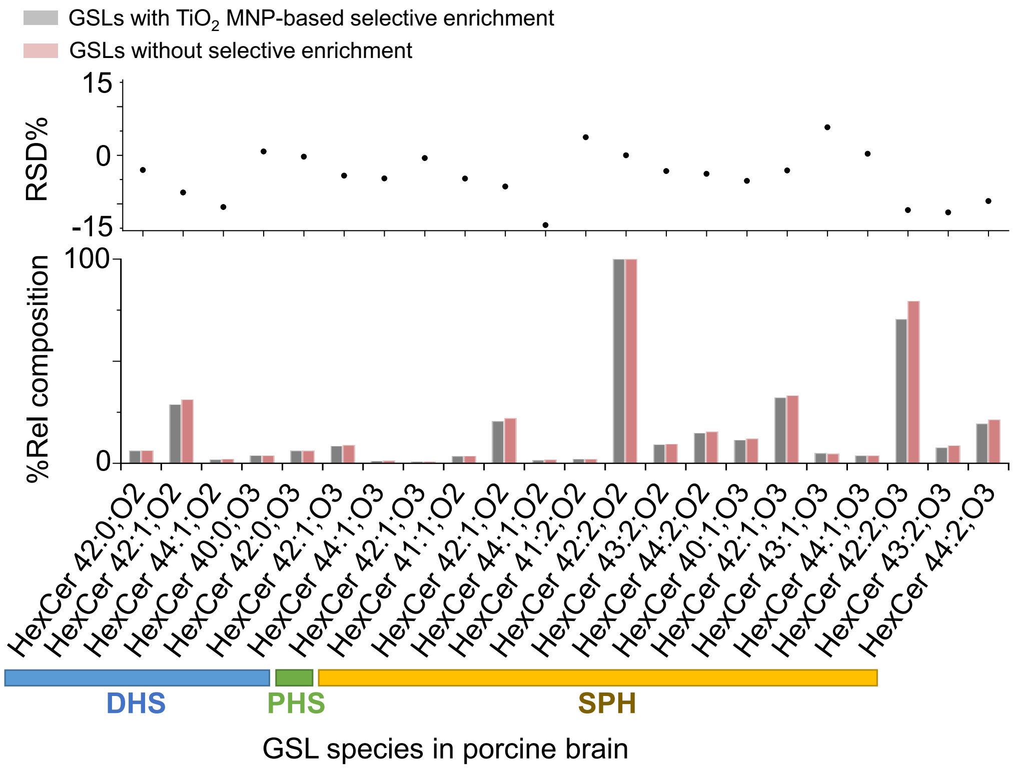

Supplementary Figure 7. Comparisons of the ion abundances of 22 neutral GSLs in porcine brain with and without selective enrichment. The 22 neutral GSLs were selected from the RPLC retention time from 15 min -30 min to minimize interference from phospholipids. Each GSL is presented as a percentage relative to the most abundant species, HexCer 42:2;O2, set at 100%. The dots represent RSDs of ion abundances of the 22 GSL species with and without selective enrichment. Source data are provided in the Source Data file.

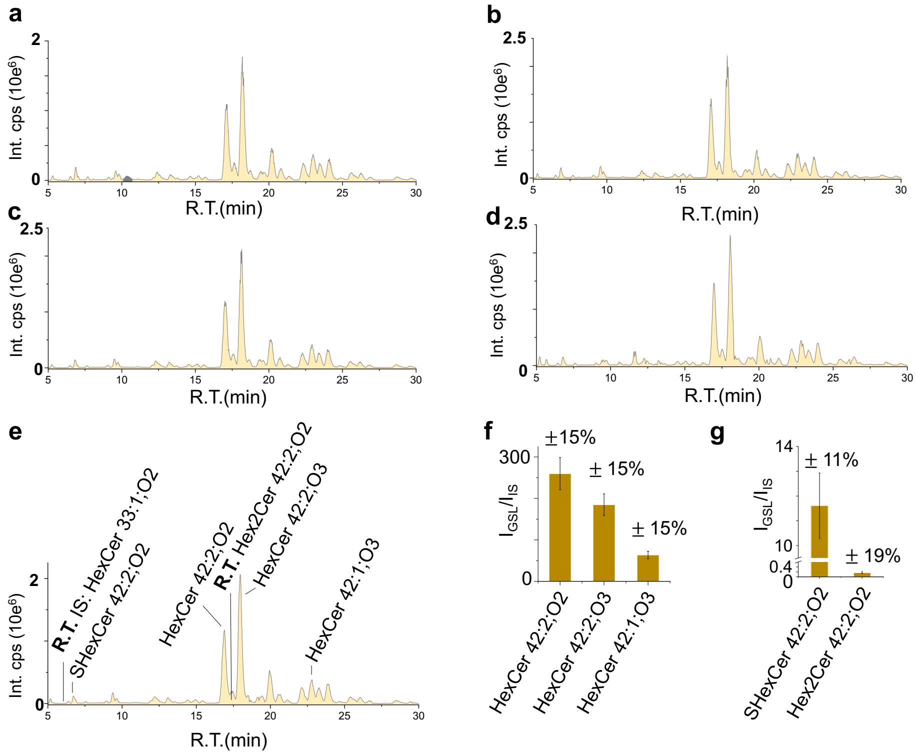

Supplementary Figure 8. Batch-to-batch reproducibility of TiO_2 MNP-based enrichment for porcine brain GSLs. a-e. Reversed-phase liquid chromatography-mass spectrometry (RPLC-MS) analysis of the equivalents of 2.5 μg of porcine brain lipid extract per injection after enrichment. f. Relative standard deviation (RSD) of the I_{GSL}/I_{IS} (100 pmol/100 μg porcine brain, $[M + H]^+$) for three major HexCer species: HexCer 42:2;O2, HexCer 42:2;O3, and HexCer 42:1;O3. g. RSD of I_{GSL}/I_{IS} for SHexCer 42:2;O2 and Hex2Cer 42:2;O2. Bars illustrate mean values ± SD of five batch-to-batch replicates. Source data are provided in the Source Data file.

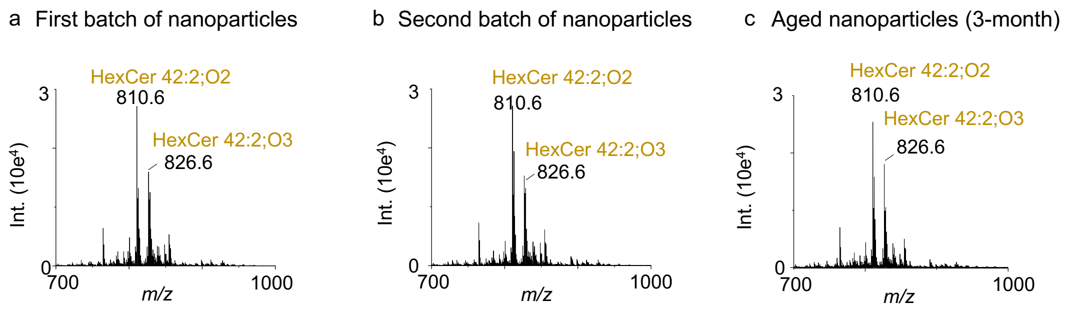

Supplementary Figure 9. Reproducibility of TiO_2 MNP-based enrichment for porcine brain GSLs using two batches of TiO_2 MNPs and aged TiO_2 MNPs. Lipid profile of 2.5 μg of porcine brain lipids per injection analyzed by RPLC-MS (RT: 5–30 min) after selective enrichment using (a-b) TiO_2 MNPs from two synthetic batches and (c) TiO_2 MNPs aged for three months.

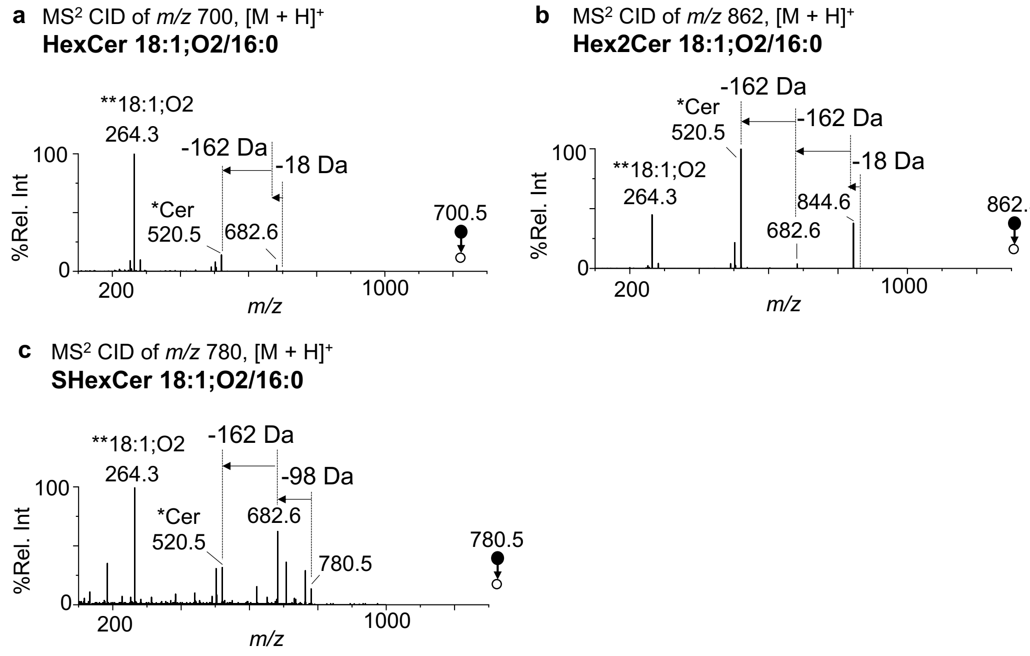

Supplementary Figure 10. MS^2 CID spectra of GSLs from porcine brain. a. HexCer 18:1;O2/16:0 (*m/z* 700.5, $[M + H]^+$), b. Hex2Cer 18:1;O2/16:0 (*m/z* 862.5, $[M + H]^+$), and c. SHexCer 18:1;O2/16:0 (*m/z* 780.5, $[M + H]^+$). The long chain base fragments and ceramide fragments are represented by d and Cer, respectively. The asterisk (*) represents sequential water loss from the LCB or Cer ions, with the number of asterisks indicating the number of water losses.

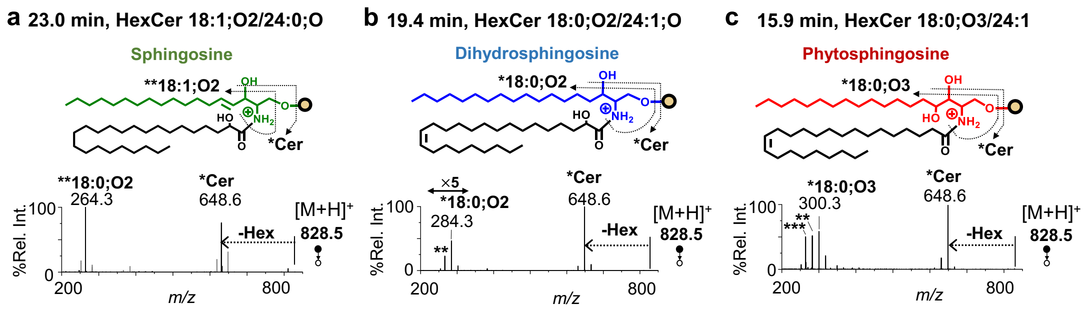

Supplementary Figure 11. MS^2 CID spectra of three LCB isomers sharing the same sum composition of HexCer 42:1;O3 ($[M+H]^+$, *m/z* 828.5) from porcine brain. a. HexCer 18:1;O2/24:0, OH (R.T. 23.0 min), b. HexCer 18:0;O2/24:1, OH (R.T. 19.4 min), and c. HexCer 18:0;O2/24:1 (R.T. 15.9 min), each showing differential LCB fragment ions (**18:1;O2, *18:0;O2, *18:0;O2) and the same ceramide ion (*Cer) resulting from the neutral loss of a hexose. The asterisk (*) represents sequential water loss form the LCB or Cer ions, with the number of asterisks indicating the number of water losses.

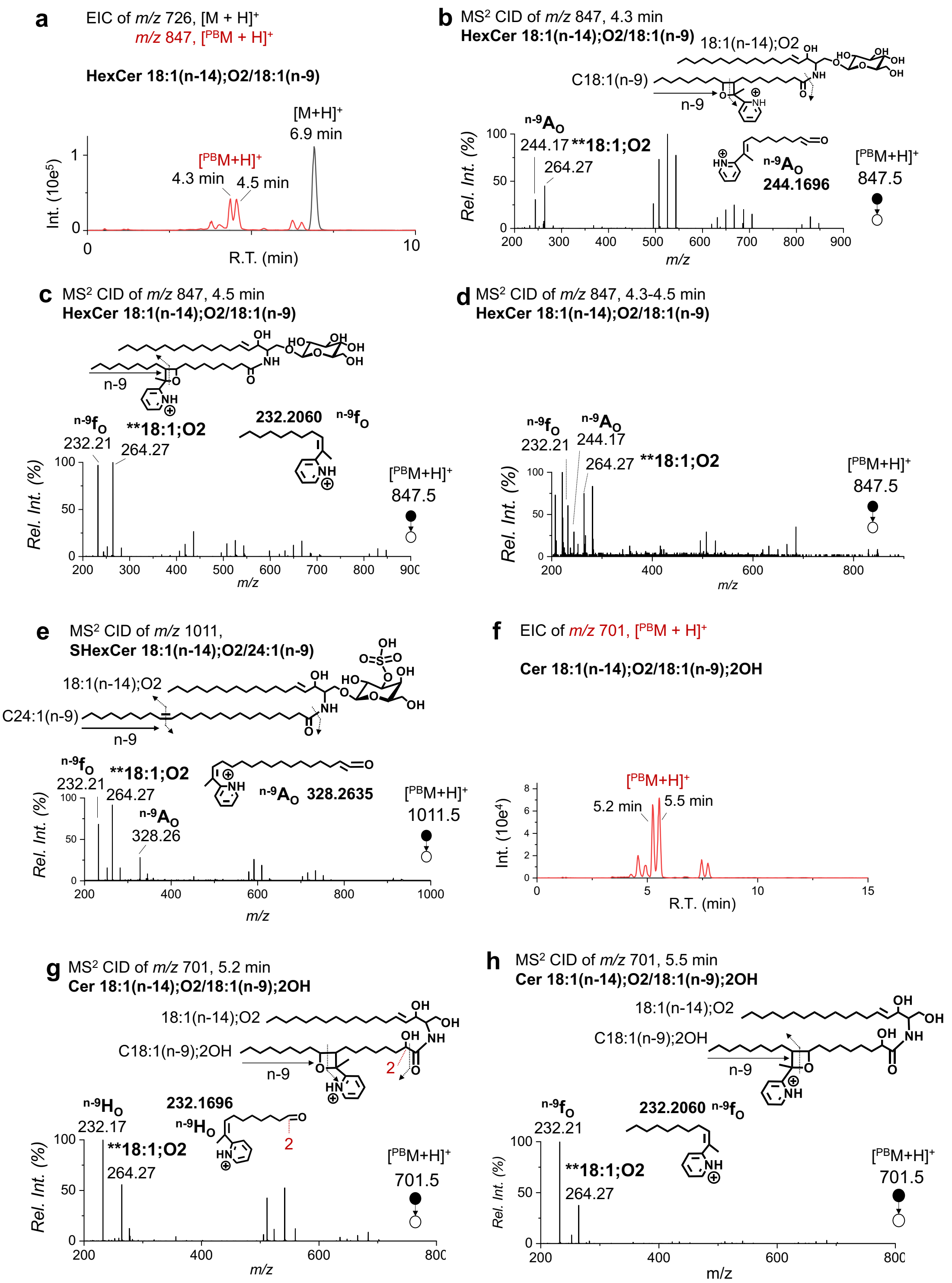

Supplementary Figure 12. Analysis of GSL standards at the C=C location level by charge-tagging PB-MS/MS. a. EIC of 5 μM of HexCer 18:1(n-14);O2/24:1(n-9) (*m/z* 726.5 $[M + H]^+$) before PB reaction and HexCer 18:1(n-14);O2/24:1(n-9) (*m/z* 847.5 $[^{PB}M + H]^+$) derivatized by 2-acpy after PB reaction. b. MS^2 CID spectra for peaks eluted at 4.3 min and c. 4.5 min in panel a. d. MS^2 CID spectrum of 5 nM of HexCer 18:1(n-14);O2/18:1(n-9) derivatized by 2-acpy. e. MS^2 CID spectrum of 10 nM of SHexCer 18:1(n-14);O2/24:1(n-9) (*m/z* 1011.5 $[^{PB}M + H]^+$) derivatized by 2-acpy after PB reaction. f. EIC of 5 μM of Cer 18:1(n-14);O2/24:1(n-9) (*m/z* 701.5 $[^{PB}M + H]^+$) derivatized by 2-acpy after PB reaction. g. MS^2 CID spectra for peaks eluted at 5.2 min and h. 5.5 min in panel f.

Supplementary Note 2: The PB products in Supplementary Figure 7 are compose of several isomers. The two regio-isomers in b-c, each lead to the formation of $^{n-9}f_O$ and $^{n-9}$Ao upon CID, are separated by RPLC. The two regio-isomers in g, h. each lead to the formation of $^{n-9}f_O$ and $^{n-9}$Ho upon CID, are separated by RPLC.

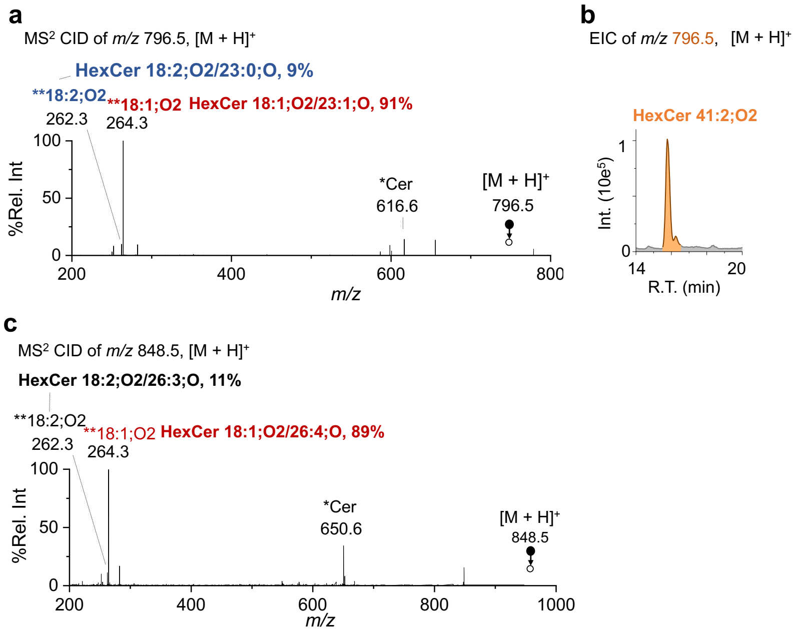

Supplementary Figure 13. Identification and relative quantitation of the chain composition isomers of porcine brain GSLs differing in degrees of unsaturation. a. MS^2 CID spectrum of HexCer 18:1;O2/23:1;O and HexCer 18:2;O2/23:0;O (*m/z* 796.5, $[M + H]^+$). b. EIC of the isomeric HexCer 41:2;O2 (*m/z* 796.5, $[M + H]^+$) as a single peak in RPLC. c. MS^2 CID spectrum of HexCer 18:1;O2/26:4;O and HexCer 18:2;O2/26:3;O (*m/z* 848.5, $[M + H]^+$). %Relative compositions of isomers are calculated by the intensity of mass peaks (**18:1;O2, **18:2;O2 ions). Source data are provided in the Source Data file.

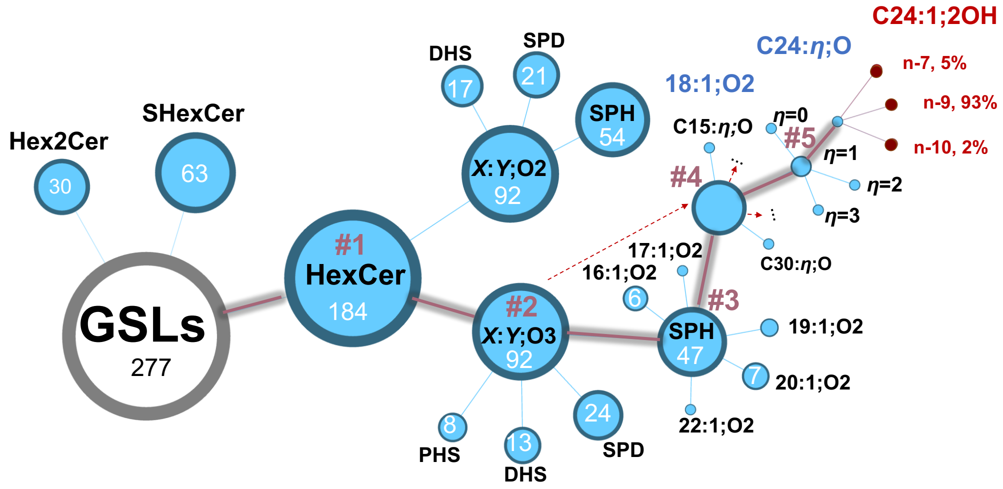

Supplementary Figure 14. Structural atlas of GSLs in human brain. The number in each circle indicates the count of GSL structures identified in that category, which is directly proportional to the size of the circle. The five-layer hierarchy is denoted by #number. Source data are provided in the Source Data file.

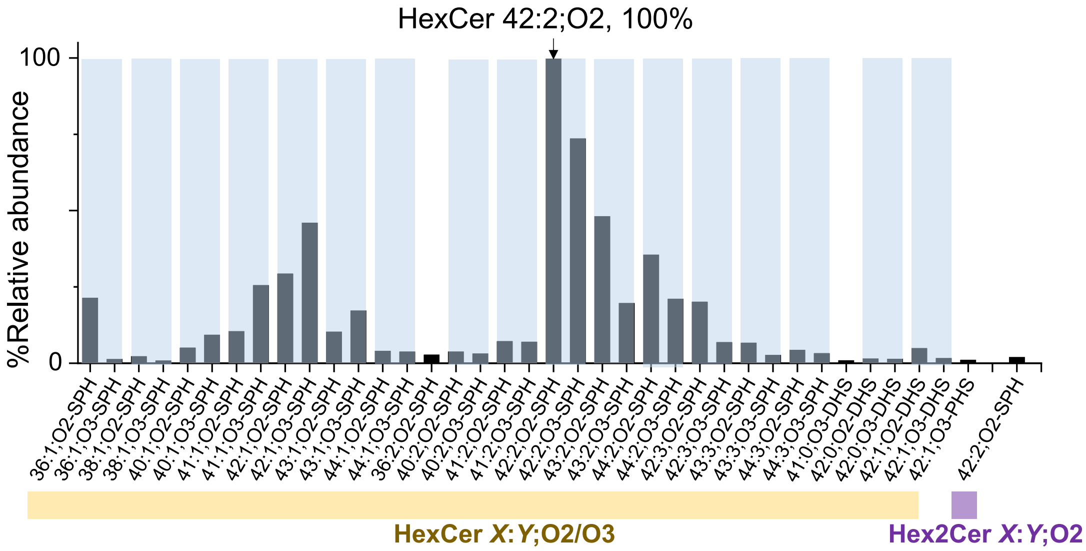

Supplementary Figure 15. The profile of 37 HexCer species and 1 Hex2Cer species with relative abundance >1% in pooled human brain sample. %Relative abundances are recorded for $[M + H]^+$. Each GSL is represented as a percentage relative to the most abundant species, HexCer 42:2;O2, which is set at 100%. The bars indicate the mean values obtained from two technically replicated measurements. Each pair of bars, highlighted with a blue shadow, represents a pair of hFA/FA analogs. Source data are provided in the Source Data file.

Supplementary Note 3: A majority of GSLs have carbon numbers in the ceramide moiety ranging from 42 to 44 and a total unsaturation of 1 to 2. GSLs containing 2OH-FA and their unmodified N-

acyl analogues were both detected. SHexCer 42:2;O2 (*m/z* 890.639, R.T. 9.1 min), the most abundant species in the SHexCer subclass, nearly coelutes with its isobaric GSL, Hex2Cer 36:1;O2 (*m/z* 890.656, R.T. 8.9 min). This demonstrates insufficient RPLC separation for quantitative analysis, as the LC peak resolution (R=1.2) is less than 1.5.

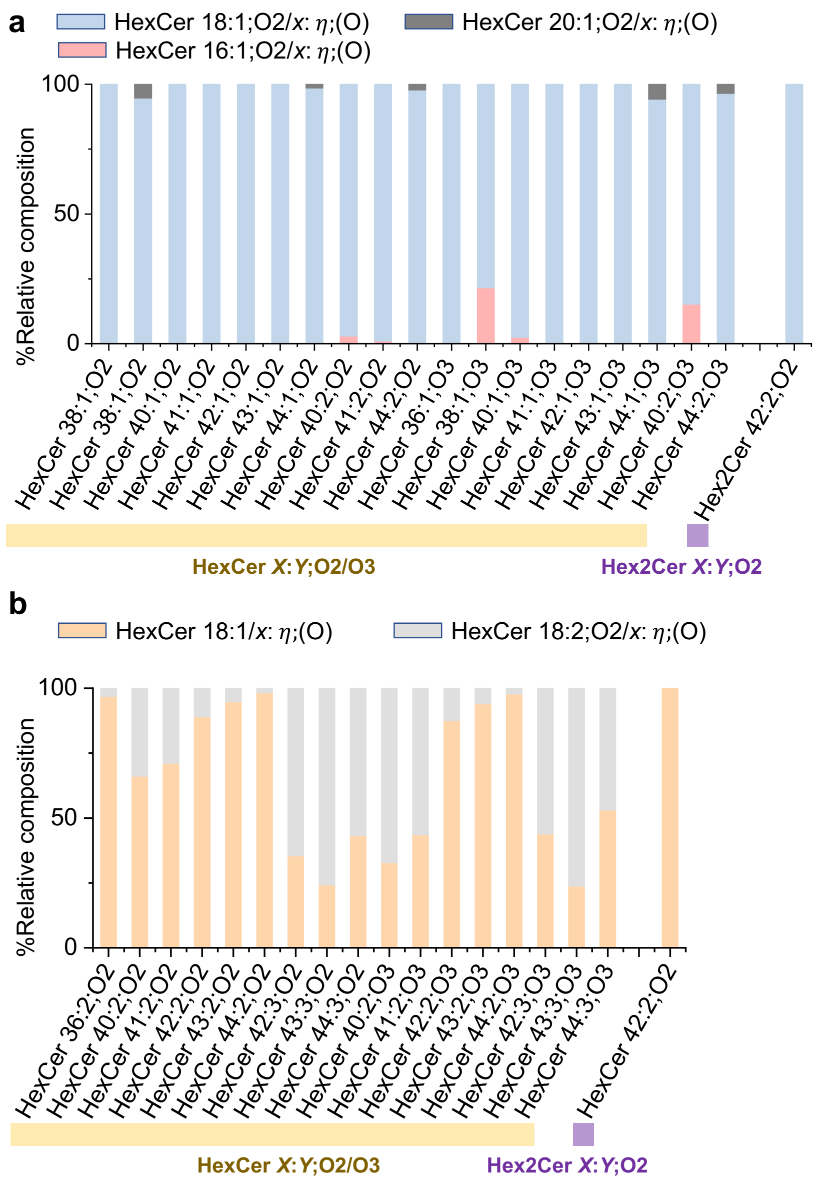

Supplementary Figure 16. The profiles of chain composition isomers of GSLs in pooled human brain. a. HexCer 16:1;O2/*x*: *η*;(O), HexCer 18:1;O2/*x*: *η*;(O), and HexCer 20:1;O2/*x*: *η*;(O), in 19 GSLs. b. HexCer 18:1;O2/*x*: *η* ;(O) and HexCer 18:2;O2/*x*: *η* ;(O), differing in degrees of unsaturation, in 17 GSLs. Bars illustrate mean values from 2 technical replicates. Source data are provided in the Source Data file.

Supplementary Note 4: In relative quantitation, chain composition isomers with a relative content below 1% are considered negligible. We detect 17 pairs of 18:2;O2/18:1;O2 and 19 groups of 16:1;O2/18:1;O2/20:1;O2 LCB isomers. Each LCB structure can produce three types of LCB ions:

*18:1;O2, which results from the loss of a water molecule; **18:1;O2, formed after losing two water molecules; and a third ion, created by the loss of both formaldehyde and a water molecule. When a GSL formula contains chain composition isomers that vary in both degrees of unsaturation and chain lengths (e.g., HexCer 18:2;O2/24:0, HexCer 17:1;O2/25:1, *m/z* 810.68), there is an overlap of the characteristic ion **17:1;O2 (*m/z* 250.25) with the third ion of 18:2;O2 LCB (*m/z* 250.25). This overlap makes it impossible to determine the relative content of 17:1;O2 using the characteristic ion **17:1;O2 (*m/z* 250.25). However, its presence can still be identified through the peak at *m/z* 268.26 (*17:1;O2), and the peak at *m/z* 238.25, corresponding to the third ion of 17:1;O2 LCB.

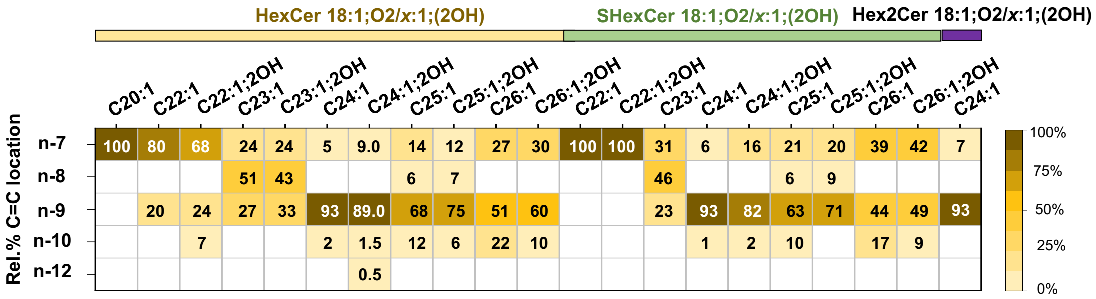

Supplementary Figure 17. Heatmap of C=C location isomers for GSLs containing monounsaturated N-acyls detected in the pooled human brain sample. The numbers shown in the figure represent the mean values of %relative composition of each C=C location isomer obtained from 3 technical replicates. Source data are provided in the Source Data file.

Supplementary Note 5: At the C=C position location level, five types of isomers (n-7, n-8, n-9, n-10, n-12) were observed for mono-unsaturated N-acyls, with n-7 and n-9 being the more abundant isomers.

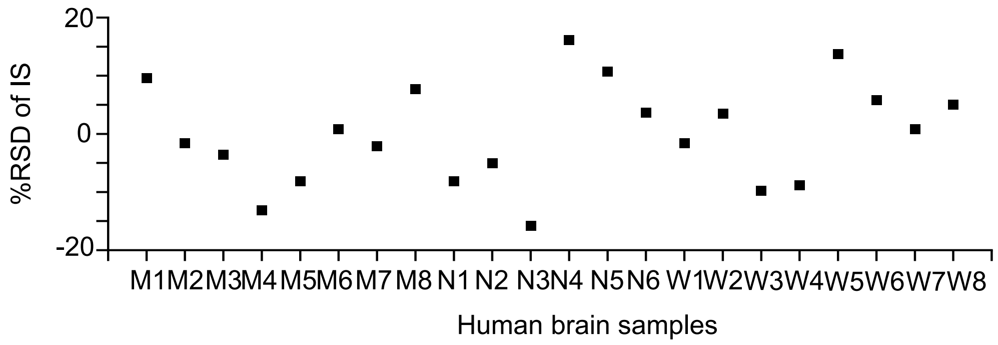

Supplementary Figure 18. RSDs of the ion abundance of IS (HexCer 18:1;O2/15:0, 200 pmol / 400 μg human brain) in 22 human brain samples after selective enrichment. Source data are provided in the Source Data file.

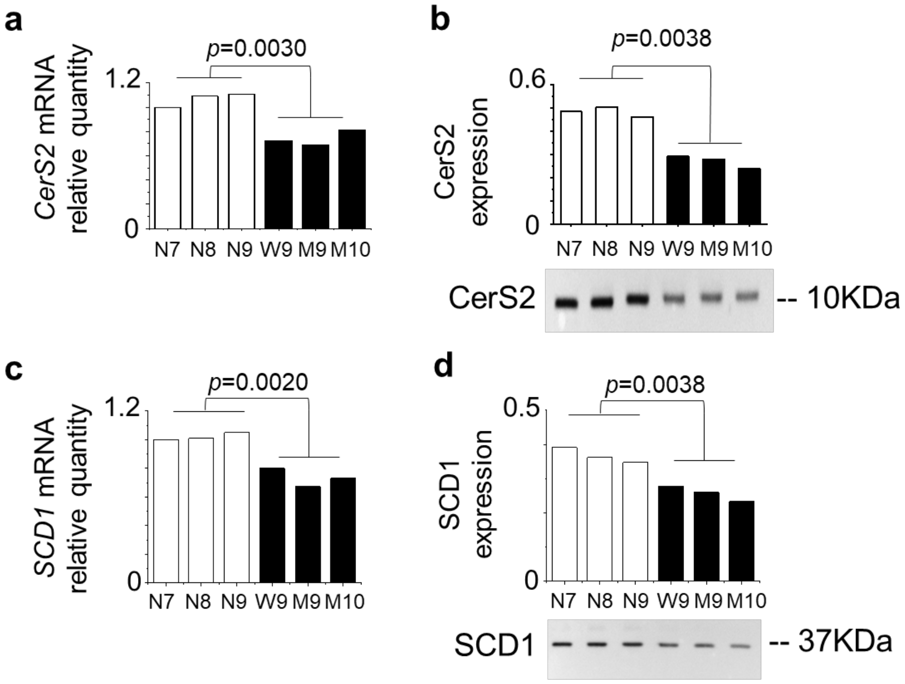

Supplementary Figure 19. Quantitative transcription analysis and Western blot analysis from six biological samples: W9, M9-M10, and N7-N9 with one independent experiment. a. Quantitative transcription analysis of Ceramide Synthase 2 (CerS2) and b. Western blot analysis of CerS2 from W9, M9-M10, and N7-N9. c. Quantitative transcription analysis of Stearoyl-CoA Desaturase-1 (SCD1) and d. Western blot analysis of SCD1 from W9, M9-M10, and N7-N9. Differences between two groups of samples were evaluated for statistical significance using the two-tailed Student's t test. Source data are provided in the Source Data file.

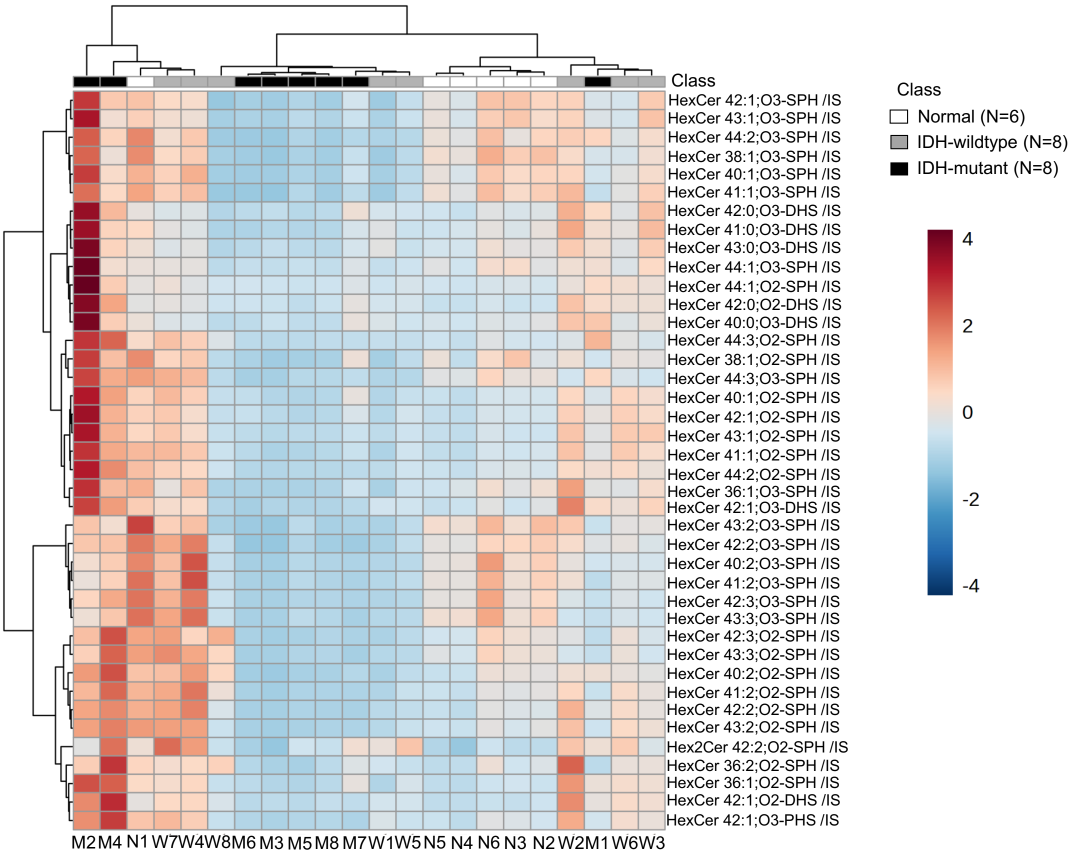

Supplementary Figure 20. Hierarchical cluster analysis using 40 species of %GSL for IDH-mutant (M1-M8), normal (N1-N6), and IDH-wildtype (W1-W8).

Supplementary Note 6: Distance measure: Euclidean; clustering method: Ward. Data analysis was conducted using online software: https://www.metaboanalyst.ca/. Colors represent the relative amounts as indicated by the color bar. Source data are provided in the Source Data file.

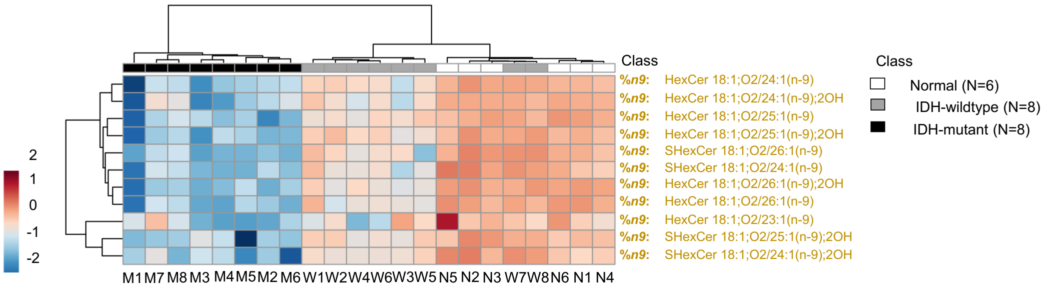

Supplementary Figure 21. Hierarchical cluster analysis using 11 groups of %n-9 for IDH-mutant (M1-M8), normal (N1-N6), and IDH-wildtype (W1-W8).

Supplementary Note 7: Distance measure: Euclidean; clustering method: Ward. Data analysis was conducted using online software: https://www.metaboanalyst.ca/. Colors represent %relative compositions as indicated by the color bar. Source data are provided in the Source Data file.

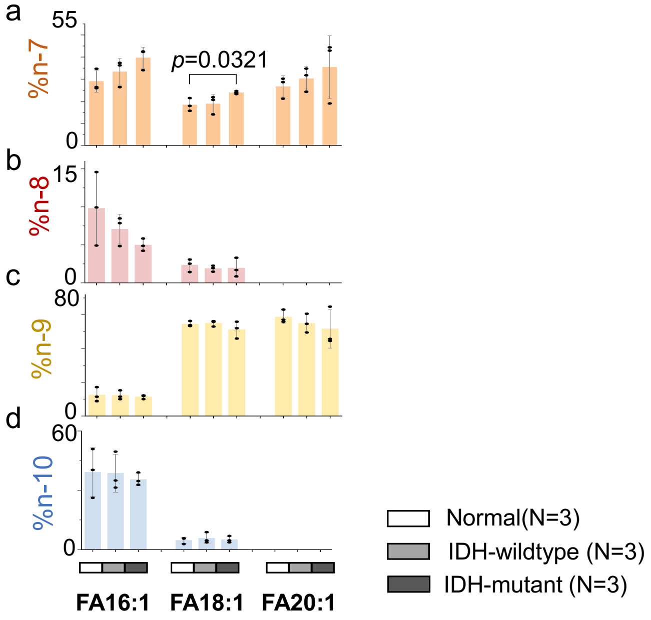

Supplementary Figure 22. Analysis of total fatty acids in human brain samples, normal sample (N2, N4, N6), IDH-mutant (M2, M3, M4), and IDH-wildtype (W2, W5, W7). %Relative compositions of a. n-7, b. n-8, c. n-9, and d. n-10 C = C location isomers in FA 16:1, FA 18:1 and FA 20:1, respectively. Bars represent mean values ±SD. Differences between two groups of samples were evaluated for statistical significance using the two-tailed Student's t test. Source data are provided in the Source Data file.

Supplementary Note 8:

Abbreviation of carbohydrate structures:
[HexCer]: Monohexosylceramides, [Hex2Cer]: Dihexosylceramides, [SHexCer]: Sulfatides
Abbreviation of long chain base types:
[SPH]: Sphingosine, [SPD]: Sphingadiene, [DHS]: Dihydrosphingosine, [PHS]: Phytosphingosine
Nomenclature of GSLs[1]:
HexCer 36:2;O2: At the sum composition level, the ceramide moiety has a total carbon number of 36 and a total degree of unsaturation of 2 across both chains. Two oxygen sites on the ceramide moiety are labeled as "O2".
HexCer 18:1;O2/16:0;O: At the chain composition level, 'O2' is used to depict the two oxygen sites in the long-chain base, as observed in sphingosine (18:1;O2) or dihydrosphingosine (18:0;O2). Similarly, 'O3' represents the three oxygen sites in the long-chain base, as seen in phytosphingosine (18:0;O3). The long-chain base has a total of 18 carbon atoms and 1 double bond. Information about the N-acyl chain is separated from the long-chain base by '/'. The N-acyl chain has 16 carbon atoms and no double bonds (C16:0). If the N-acyl chain possesses an oxygen sites, 'O' will be appended after it (C16:0;O).

HexCer 18:1(n-14);O2/18:1(n-9);2OH: If the locations of the C=C bonds are confirmed,

the notation "n-x" will be added after each chain. For example, "n-14" signifies that the double bond is located at the 14th carbon from the alkyl end in long chain base, while "n-9" indicates the double bond at the 9th carbon from the alkyl end in N-acyl chain. Additionally, "2OH" indicates that the position of the hydroxyl group is confirmed at carbon 2 in the N-acyl chain.

Supplementary Table 1. The table lists the amounts of two phospholipids removed from porcine brain through selective enrichment and the deviation in the amounts of two HexCers in porcine brain through selective enrichment with different batches of TiO_2 MNPs.

**Phospholipids removed from porcine brain (2.5 μg each injection)**				
**Component Name**	**$[M+H]^+$, *m/z***	**Intensity with selective enrichment**	**Intensity without selective enrichment**	**%Removal**
PC 36:1	788.6	567059.0454	196222865	99.71
PC 34:1	760.6	681426.0008	266685861	99.74
**Selective enrichment of porcine brain HexCers with two batches of nanoparticles (5 μg each injection)**				
**Component Name**	**$[M+H]^+$, *m/z***	**Intensity with first batch of nanoparticles**	**Intensity with second batch of nanoparticles**	**RSDs for different batches** (%)
HexCer 42:2;O2	810.6	27109.53208	27136.03838	-0.10
HexCer 42:2;O3	826.6	15944.81822	15230.78309	4.58
**Selective enrichment of porcine brain HexCers with freash and aged nanoparticles (5 μg each injection)**				
**Component Name**	**[M+H]+, *m/z***	**Intensity with fresh nanoparticles**	**Intensity with aged nanoparticles (3-month)**	**RSDs for aged batches** (%)
HexCer 42:2;O2	810.6	27136.03838	25409.22158	-6.57
HexCer 42:2;O3	826.6	15230.78309	18043.60966	16.91

Supplementary Table 2. Gene-specific primers for *FA2H*, *CerS2*, *SCD1*.

**Target Name**	**Primer**	
CerS2	*CerS2-F*	GATTACTTCTGGTGGGAACGTCT
	CerS2-R	GACACGTCCATCTCGGTCT
FA2H	*FA2H-F*	CCGCCTCTTCCACTCAGACC
	FA2H-F	AATGACGTGAAGAGTCGGACGTT

SCD1	*SCD1-F*	CTACAAACCTGGCTTGCTG
	SCD1-R	AAGAAAGTGGCAACGAACACA

Supplementary Table 3. The table lists 26 GSL structures profiled by PB-MS/MS analysis in individual human brain samples. The data includes the *m/z* of [^{PB}M + H]$^+$, R.T.s, diagnostic ions for C=C and 2OH location.

GSL strutures at chain composition level	[^{PB}M+H]$^+$, *m/z*	R.T., min	Diagnostic ions, f_O, A_O or H_O, *m/z*	GSL structures at C=C & 2OH location level (Assuming trans C-4 C=C in SPH)
HexCer 18:1;O2/26:1,2OH	975.76	14.4	204.17,372.29;	HexCer 18:1;O2(n-14)/26:1(n-7) ;2OH
			232.21,344.29;	HexCer 18:1;O2(n-14)/26:1(n-9) ;2OH
ShexCer 18:1;O2/26:1	1039.72	8.17	204.17,384.29;	SHexCer 18:1;O2(n-14)/26:1(n-7)
			232.21,356.29;	SHexCer 18:1;O2(n-14)/26:1(n-9)
SHexCer 18:1;O2/25:1	1025.71	6.86	204.17,370.30;	SHexCer 18:1;O2(n-14)/25:1(n-7)
			232.21,342.27;	SHexCer 18:1;O2(n-14)/25:1(n-9)
HexCer 18:1;O2/25:1	945.75	13.3	204.17,370.30;	HexCer 18:1;O2(n-14)/25:1(n-7)
			218.19,356.29;	HexCer 18:1;O2(n-14)/25:1(n-8)
			232.21,342.27;	HexCer 18:1;O2(n-14)/25:1(n-9)
			246.22,328.26;	HexCer 18:1;O2(n-14)/25:1(n-10)
HexCer 18:1;O2/25:1,2OH	961.75	12.3	204.17,358.30;	HexCer 18:1;O2(n-14)/25:1(n-7) ;2OH
			232.21,330.27;	HexCer 18:1;O2(n-14)/25:1(n-9) ;2OH
HexCer 18:1;O2/24:1,2OH	947.68	10.4	204.17,344.29;	HexCer 18:1;O2(n-14)/24:1(n-7) ;2OH
			232.21,316.26;	HexCer 18:1;O2(n-14)/24:1(n-9) ;2OH
SHexCer 18:1;O2/24:1,2OH	1027.69	5.4	204.17,344.29;	SHexCer 18:1;O2(n-14)/24:1(n-7) ;2OH
			232.21,316.26;	SHexCer 18:1;O2(n-14)/24:1(n-9) ;2OH
HexCer 18:1;O2/24:1	931.73	11.3	204.17,356.29;	HexCer 18:1;O2(n-14)/24:1(n-7)
			232.21,328.26;	HexCer 18:1;O2(n-14)/24:1(n-9)
			246.22,314.24;	HexCer 18:1;O2(n-14)/24:1(n-10)
SHexCer 18:1;O2/24:1	1011.69	5.6	204.17,356.29;	SHexCer 18:1;O2(n-14)/24:1(n-7)
			232.21,328.26;	SHexCer 18:1;O2(n-14)/24:1(n-9)
HexCer 18:1;O2/23:1	917.72	9.5	204.17,342.27;	HexCer 18:1;O2(n-14)/23:1(n-7)
			218.19,328.26;	HexCer 18:1;O2(n-14)/23:1(n-8)
			232.21,314.24;	HexCer 18:1;O2(n-14)/23:1(n-9)
HexCer18:1;O2,26:1,2OH	959.77	15.6	204.17,384.29;	HexCer 18:1;O2(n-14)/26:1(n-7)
			232.21,356.29;	HexCer 18:1;O2(n-14)/26:1(n-9)

Supplementary methods

Materials.

Iron chloride hexahydrate ($FeCl_3 \cdot 6H_2O$) was acquired from 3A Materials. Anhydrous ethanol (EtOH), ethylene glycol, chloroform, and ammonium hydroxide were purchased from Guoyao Chemical Reagent Company (Shanghai, China). Ammonium bicarbonate, titanium (IV) butoxide, analytical-grade formic acid, hydrochloric acid, methanol, acetonitrile, and isopropyl alcohol were purchased from Fisher Scientific Company (Ottawa, ON, Canada). Sodium formate and charge-tagging PB reagents 2-Acetylpyridine (2-acpy) were obtained from Bidepharm (Shanghai, China). All other reagents were of analytical grade and procured from commercial suppliers.

HexCer 18:1(n-14);O2/18:1(n-9), SHexCer 18:1(n-14);O2/24:1(n-9), Cer 18:1(n-14);O2/18:1(n-9), Cer 18:1(n-14);O2/18:1(n-9);2OH, PE 16:0/18:1, SM 18:1(n-14);O2/12:0, PC 16:0/18:1, and brain polar lipid extract (Porcine) were purchased from Avanti (Alabaster, AL, USA). SHex2Cer 18:1;O2/18:0, Hex3Cer 18:1;O2/18:0, Gb4 18:1;O2/24:0, HexCer 18:1(n-14);O2/15:0, Hex2Cer 18:1(n-14);O2/17:0, SHexCer 18:1(n-14);O2/17:0 were obtained from Cayman (Ann Arbor, MI, USA). The FA2H polyclonal antibody (1542-1-AP) was sourced from Proteintech (Wuhan, Hubei, China), and the CerS2 Antibody (rabbit polyclonal, A303-193A-T) was acquired from BETHYL (Hamburg, Germany). Anti-SCD1 (Catalog No. ab236868, clone EPR21963) was acquired from Abcam (Cambridge, UK). Protein extraction buffer (MDL91201), cocktails of protease inhibitors (MD912893), BCA Protein Assay Kit (MD913053), SDS-PAGE Kit (MD911919), Anti-actin (MD6553), and horseradish peroxidase (HRP, MD912565, Polyclonal Goat Anti-Rabbit IgG) were obtained from Medical Discovery

Leader (Beijing, China). The protein standard marker (26617) was purchased from Thermo (Waltham, MA, USA).

Synthesis of TiO_2 MNPs.

The magnetic nanocores were constructed following a procedure reported previously[2-4]. Briefly, a solution of anhydrous sodium acetate (7.2 g) and $FeCl_3·6H_2O$ (2.7 g) as a ferric source in 150 mL of ethylene glycol was vigorously stirred to obtain a transparent solution. The mixture was then sealed in a Teflon-lined, stainless-steel autoclave and heated at 200 °C for 16 hours. The resulting product, settling at the bottom of the autoclave, was washed three times with water and ethanol under ultrasonic conditions, and subsequently dried at 50 °C to obtain magnetic core. Magnetic force was utilized to separate the product from various solvents at each step. 80 mg of dehydrated magnetic core were dispersed in 195 mL of ethyl alcohol. Then, 0.72 mL of concentrated ammonia solution (28 wt%) was injected into the solution. The mixed solution, containing 1.6 mL of titanium (IV) butoxide and 5 mL of ethyl alcohol, was added dropwise into the reaction system. The system was kept at 45°C for 24 hours. The Fe_3O_4@TiO_2 were washed with ethyl alcohol four times and dehydrated in a vacuum drying oven. The Fe_3O_4@TiO_2 were then calcined at 400°C for 2 hours and stored at room temperature. For each batch, ~1 g of TiO_2 MNPs were prepared and used for experiments spanning three months.

Nanomaterial characterization

Fe_3O_4 nanocore, Fe_3O_4@TiO_2, and TiO_2 MNPs were analyzed by Transmission Electron Microscopy (TEM). The TEM images were acquired by FEI Tecnai G2 20

transmission electron microscopy. TiO_2 MNPs were sent for Scanning Electron Microscopy (SEM) analysis. The SEM images and mapping of elements were acquired by Quanta 450 FEG Thermal Field Emission Scanning Electron Microscope. Nitrogen adsorption and desorption isotherms were measured using Quantachrome IQ. The Brunauer–Emmett–Teller (BET) method was utilized to calculate the specific surface areas (SBET) using adsorption data. By using the Barrett–Joyner–Halenda (BJH) model, the pore volumes and pore size distributions were derived from the adsorption branches of the isotherms. Zeta potential measurements were carried out on a Nano ZS90 zeta analyzer (Malvern Instruments Ltd.). The chemical compositions of TiO_2 MNPs were detected through X-ray photoelectron spectra (XPS, Thermo ESCALAB 250Xi, Al Kα X-ray source).

Sample preparation

The diagnosis of isocitrate dehydrogenase genetic (IDH) mutation in human brain tissue samples was confirmed using Immunohistochemistry staining and Polymerase Chain Reaction Sequencing, specifically targeting Isocitrate Dehydrogenase 1 R132 and Isocitrate Dehydrogenase 2 R172.

Lipid extraction was performed based on a modified Folch method[5]. Briefly, 25 mg of human brain tissue sample was placed in a 10 mL-centrifuge tube. 1 mL of deionized water were added. The tissue sample was homogenized by a handheld homogenizer (Jingxin Technology) at 40,000 Hz for 5 min. 1 mL of methanol (MeOH) and 2 mL chloroform were further added and the tube was agitated for 10 min. The mixture was centrifuged at 12960 x g for 10 min and the bottom layer was extracted and transferred

to another tube. The above extraction process was repeated once. The bottom layers from the two extractions process were combined and dried under nitrogen flow. Then, 1 mL of MeOH was added to disperse the extracts and stored at -20 °C for further analysis. The internal standard (HexCer 18:1;O2/15:0, 200 pmol) was added to the solution of lipid extract from 400 μg of human brain tissue.

Following a reported method[6], total fatty acids (FAs) were hydrolyzed from glycerol lipids. An equivalent of lipids from 40 μg of human brain tissue was saponified in 500 μL of MeOH:15% KOH (50/50, v/v) at 37 °C for 30 min. The solution was subsequently acidified with 1 M HCl (1 mL). The hydrolyzed lipids were extracted twice with 1.5 mL isooctane each time. The organic layer was then collected, dried under nitrogen, and redissolved in an aliquot of 200 μL of MeOH for further derivatization.

Selective enrichment of GSLs

For the enrichment of GSLs from an artificial lipid mixture, 5 nM (0.008 μg) of each GSL (HexCer 18:1;O2/18:1, HexCer 18:1;O2/15:0, HexCer 18:1;O2/24:1, HexCer 18:1;O2/18:0;2OH, Hex2Cer 18:1;O2/17:0), along with 25 μM (8 μg) of phosphatidylethanolamine (PE 16:0/18:1), 50 μM of sphingomyelin (SM 18:1;O2/12:0, 13 μg), and 50 μM of phosphatidylcholine (PC 16:0/18:1, 16 μg), were mixed with 5 mg of TiO_2 MNPs in 400 μL of loading buffer (94% acetonitrile and 6% aqueous NH_3, v/v) and agitated for 1 hour at room temperature. TiO_2 MNPs were then separated by a magnet and washed by washing buffer (MeOH/aqueous NH_3, 96/4, v/v, 20 mM NH_4HCO_3) twice (2 × 400 μL). Elution was performed with 400 μL of eluting buffer (95% MeOH and 5% formic acid, v/v) with 2 hours of agitation. The ammonia solution

used above is 28 wt%, and the formic acid is 88 wt%. The separated GSL standards were reconstituted in 100 μL ACN, representing a 4-fold enrichment compared to the volume of the loading buffer. The same enrichment process was implemented with different concentrations of GSLs (12.5 nM, 500 nM, 1250 nM for each GSL standard). To assess the recovery of GSLs under various concentrations, we also prepared a series of GSL standard mixtures by combing the five GSL standards at three different concentrations: 20 nM, 50 nM, and 5000 nM. Both the enriched GSLs and the GSL standard mixtures were then subjected to mass spectrometry (MS) analysis. For example, the recovery of GSLs at a 5 nM concentration was calculated by comparing the intensity of enriched GSLs (primarily at a 5 nM concentration with subsequent 4-fold enrichment) to the standard GSL mixtures (with a concentration of 20 nM GSLs). The recovery of sulfatides (at concentrations of 50 nM, 125 nM, 1250 nM) was also tested and analyzed using the same procedure above.

For the enrichment of GSLs from porcine brain lipid extracts, 100 μg of porcine brain lipids were mixed with 5 mg of TiO_2 MNPs, 100 pmol of IS HexCer 18:1;O2/15:0, and 400 μL of loading buffer for 1 h agitation. TiO_2 MNPs were separated by magnet and washed by washing buffer for two times. GSLs were released by 2 h agitation in eluting buffer. The separated GSLs were reconstituted in 100 μL of acetonitrile.

Pooled human brain lipid extracts were created by blending lipids from one IDH-mutant, one IDH-wildtype, and one normal sample in a 1:1:1 mass ratio. For GSL enrichment from human brain lipid extracts, an equivalent amount of lipids (either pooled or individual human brain lipid extracts) from 400 μg of human brain tissue was mixed

with 5 mg of TiO_2 MNPs, 200 pmol of IS HexCer 18:1;O2/15:0, and 400 μL of loading buffer for 1 h agitation. TiO_2 MNPs were separated by magnet and washed by washing buffer for two times. GSLs were released by 2 h agitation in eluting buffer. The separated GSLs were reconstituted in 100 μL of acetonitrile (ACN).

Offline PB derivatization.

The PB derivatization was performed using a homemade flow microreactor[7]. A GSL standard or ceramide standard at a concentration of 5 μM was dissolved in a 100 μL solvent (ACN/H_2O, 5/1, v/v) containing 10 mM 2-acpy reagent. The solution was deoxygenated with nitrogen for 10 minutes and then injected into the flow microreactor for 15 seconds of UV irradiation (~254 nm).

GSLs enriched from 100 μg of porcine brain lipids or lipid extract from 400 μg of human brain tissue were mixed with 10 mM 2-acpy in a 100 μL system (ACN/H_2O, 5/1, v/v) for a 15-second PB reaction.

FA extracts that were dried from a 50 μL stock solution and 10 mM PB reagent were dissolved in 100 μl ACN. The solution was then injected into the flow microreactor for 25 seconds of UV irradiation (∼254 nm).

RPLC-MS/MS

RPLC conditions for GSLs

All LC separations were performed on a 20ADLC system (SHIMADZU, Tokyo, Japan) connected to an X500R QTOF mass spectrometer (SCIEX, Toronto, Canada). Intact GSLs and their PB products were analyzed using an XBridge BEH C18 Column (130Å,

2.5 µm, 2.1 mm x 100 mm, Waters, Milford, MA, USA). Mobile phase A consisted of ultrapure water with 10 mM of ammonium formate and 0.1% formic acid as additives. Mobile phase B comprised ACN and IPA in a 9:1 (v:v) ratio. The mobile phases were used for gradient elution 1 and 2.

Gradient elution 1 (36 min)

The gradient elution was modified from an isocratic elution (acetonitrile/water = 95:5, v/v) reported by Sugawara et al[8]. Initially, mobile phase B was set at 90% and maintained for 1 minute. Then, it was increased to 93% at 10 minutes, held at 93% for 2 minutes, and subsequently elevated to 99% at 32 minutes. It remained at 99% for 3.5 minutes and was then reduced to 90% at 36 minutes. The flow rate was set to 0.5 mL/min, and the column temperature was maintained at 40 °C. Gradient elution 1 was utilized for intact GSL separation in porcine brain, pooled human brain, and individual human brain samples. Additionally, Gradient elution 1 was employed for PB-derivatized GSL separation in individual human brain samples.

Gradient elution 2 (49 min)

Initially, mobile phase B was set at 85% and held for 1 minute, then increased to 88% at 10 minutes, maintained at 88% for 2 minutes, and raised to 94% at 32 minutes. It remained at 94% for 3 minutes, was elevated to 99% at 36.5 minutes, held at 99% for 11.5 minutes, and finally reduced to 85% at 49 minutes. The flow rate was 0.45 mL/min, and the column temperature was set at 40 °C. Gradient elution 2 was applied for PB-

derivatized GSL separation in porcine brain and pooled human brain samples.

MS parameters for GSLs

MS analysis employed data collection modes of targeted MS/MS and Sequential Window Acquisition of All Theoretical Mass Spectra (SWATH). The MS parameters were optimized as follows: ESI voltage at 5000 V, curtain gas at 30 psi, interface heater temperature at 400 °C, nebulizing gas 1 and gas 2 at 30 psi, declustering potential at 80 V. ESI positive calibration solution X500 (SCIEX, Toronto, CA) was used to perform calibration for *m/z*. The isolation windows in SWATH mode and collision energy are provided in Supplementary data 7.

Targeted MS/MS

Each targeted MS/MS method (with a 0.7 Da width and 1s accumulation time, 10.0 s total scan time) involved a list of 10 precursor ions (subject to mode restriction) for the chain composition analysis of porcine brain GSLs. The precursor ions were calculated based on theoretical m/z values of GSLs ($[M + H]^+$), considering different GSL subclasses (HexCer, Hex2Cer, SHexCer), degrees of unsaturation (0-4), carbon numbers in ceramide moiety (32-48), and additional hydroxylation (0-1). The mass spectra were recorded in the range from *m/z* 100 to 1000 in positive electrospray mode. The identified precursor ions were documented in the GSL library. Targeted MS/MS method was also used for C=C location analysis of unsaturated GSLs in porcine brain and pooled human brain samples. The mass spectra were recorded in the range from

m/z 100 to 1300 in positive electrospray mode. The identified precursor ions were documented in the ^{PB}GSL library.

SWATH data acquisition

The SWATH data acquisition mode was employed to enhance the data collection throughput once the GSL and ^{PB}GSL libraries were established by targeted MS/MS. The SWATH method for chain composition analysis included an MS scan covering the range from *m/z* 500 to 1300, accompanied by 79 MS/MS scans (*m/z* 100 -1000) utilizing various isolation windows (1 Da width, 0.1s accumulation time, 8.4s total scan time). This approach was based on the validated GSL library in targeted MS/MS and included 3 technical replicates. SWATH acquisition mode was utilized to analyze pooled human brain sample and individual human brain samples with 2 technical replicates.

The SWATH method for C=C location analysis included an MS scan covering the range of *m/z* 500 to 1500. Additionally, it included 16-23 MS/MS scans (*m/z* 100 -1000) with various isolation windows (1 Da width, 0.1s accumulation time), profiling GSLs with monounsaturated N-acyls in porcine brain and pooled human brain, carried out with 3 technical replicates. SWATH acquisition mode was also utilized for profiling of C=C location isomers of GSLs with monounsaturated N-acyls in individual human brain samples, with 2 technical replicates. Since some human brain samples have relatively lower GSL abundance compared to others, we injected them with 3-4 technical replicates to ensure signal stability. The *m/z* $[^{PB}M + H]^+$, R.T.s, diagnostic ions are listed

in Supplementary Table 3.

Analysis of total FA

The LC method followed our previous report[6]. A C18 column (150 mm × 3.0 mm, 2.7 μm, Sigma-Aldrich, MO) was used to separate derivatized total fatty acids. Mobile phase A comprised H_2O and ACN in a 4:6 (v/v) ratio and 20 mM ammonium formate as additive. Mobile phase B comprised IPA and ACN in a 4:6 (v/v) ratio and 0.2% formic acid as additive. The flow rate was set at 0.45 mL/min. The chromatographic gradient was as follows: 30% B at 0–0.75 min, 30%–45% B at 0.75–2 min, 45%–52% B at 2–2.5 min, 52%–58% B at 2.5–4 min, 58%–66% B at 4–5.5 min, 66%–70% B at 5.5–7 min, 70%–75% B at 7–9 min, 75%–97% B at 9–10 min, 97% B at 10–13 min, 30% B at 13.1–15 min. The flow rate was set to 0.5 mL/min, and the column temperature was maintained at 55 °C. The MS parameters were optimized as follows: ESI voltage, 4500 V; curtain gas, 35 psi; interface heater temperature, 400 °C; nebulizing gas 1 and gas 2, 30 psi; declustering potential, 100 V. The SWATH method included an MS scan covering the range of *m/z* 100 to 500. Additionally, it included 23 MS/MS scans (*m/z* 100 -500) with various isolation windows (1 Da width, 0.1s accumulation time) and carried out with 3 technical replicates. The isolation windows in SWATH mode and collision energy are provided in Supplementary Data 7.

Data analysis

Data analysis was performed using SCIEX OS (version 3.0.03339). GSL identification was conducted manually, considering RPLC retention time, accurate *m/z*, and

fragmentation rules. All mass peaks with a signal-to-noise ratio (S/N) > 3 were utilized for identification.

The GSL species with relative ion abundances exceeded 1% of the most abundant GSL in MS^1 were chosen for relative quantitation at different structural levels. Regarding %hFA, the selected GSLs should be commonly represented in 85% of individual human brain samples. Regarding %SPD, only those having %SPD higher than 5% were considered. For %C24, only those having %24 higher than 0.5% were considered. Given the high sensitivity of C=C location isomers, no additional criterium was used for their relative quantitation.

Protein extraction and Western blotting

For western blot and qRT-PCR analysis, we used samples from three normal tissues and three glioma tissues (two IDH-mutant and one IDH-wildtype). Protein extraction and quantification were performed on each 50 mg wet weight human brain tissue. The proteins were separated using SDS-PAGE (BIO-Rad, Hercules, CA, USA) and transferred to nitrocellulose membranes. These membranes were blocked using 5% non-fat milk and probed with the designated primary antibodies (diluted at 1:500–1000). After incubation with horseradish peroxidase-conjugated secondary antibodies (diluted at 1:5000), the proteins were visualized through chemiluminescence, and the signals (BIO-Rad, Hercules, CA, USA) were quantified.

RNA extraction and qRT-PCR

Total RNAs were isolated using TRizol Reagent (Thermo Fisher Scientific) and reverse transcribed was proceeded with reverse transcribed kit superscript III (Invitrogen). The

gene-specific primers for *FA2H*, *CerS2*, *SCD1* were listed in Supplementary Table 2. qRT-PCR ran on a StepOne™ Real-Time Fluorescent Quantitative PCR System (Applied biosystems, Carlsbad, CA, USA).

Supplementary References

1. Liebisch, G. et al. Update on LIPID MAPS classification, nomenclature, and shorthand notation for MS-derived lipid structures. *Journal of Lipid Research* **61**, 1539-1555 (2020).
2. Qi, D., Lu, J., Deng, C. & Zhang, X. Magnetically Responsive Fe3O4@C@SnO2 Core-Shell Microspheres: Synthesis, Characterization and Application in Phosphoproteomics. *The Journal of Physical Chemistry C* **113**, 15854-15861 (2009).
3. Xu, X. et al. Synthesis of Magnetic Microspheres with Immobilized Metal Ions for Enrichment and Direct Determination of Phosphopeptides by Matrix-Assisted Laser Desorption Ionization Mass Spectrometry. *Advanced Materials* **18**, 3289-3293 (2006).
4. Lu, J., Wang, M., Deng, C. & Zhang, X. Facile synthesis of Fe3O4@mesoporous TiO2 microspheres for selective enrichment of phosphopeptides for phosphoproteomics analysis. *Talanta* **105**, 20-27 (2013).
5. Folch, J., Lees, M. & Sloane Stanley, G.H. A simple method for the isolation and purification of total lipides from animal tissues. *The Journal of biological chemistry* **226**, 497-509 (1957).
6. Zhao, J., Fang, M. & Xia, Y. A liquid chromatography-mass spectrometry workflow for in-depth quantitation of fatty acid double bond location isomers. *Journal of Lipid Research* **62**, 100110 (2021).
7. Zhao, J. et al. Next-Generation Paternò–Büchi Reagents for Lipid Analysis by Mass Spectrometry. *Analytical Chemistry* **92**, 13470-13477 (2020).
8. Sugawara, T., Aida, K., Duan, J. & Hirata, T. Analysis of Glucosylceramides from Various Sources by Liquid Chromatography-Ion Trap Mass Spectrometry. *Journal of Oleo Science* **59**, 387-394 (2010).
