# Supplementary material for: Illuminating the dark space of neutral glycosphingolipidome by selective enrichment and profiling at multi-structural levels
Source: Nat Commun. 2024 Jul 4;15:5627. doi: 10.1038/s41467-024-50014-8 (PMC11224418; doi:10.1038/s41467-024-50014-8)
Supplement: Supplementary file 3 — Description of Additional Supplementary Files [file 41467_2024_50014_MOESM3_ESM.pdf]

Description of Additional Supplementary Files

File Name: Supplementary Data 1

Description: The table lists 86 GSLs detected in RPLC-MS spectrum from porcine brain. It includes data on the m/z of [M + H]+, mass errors, R.T., and % relative abundances.

File Name: Supplementary Data 2

Description: The table includes the retention patterns of GSLs in RPLC. It includes data of the R.T. of GSLs with varying carbon numbers, degrees of unsaturation, and LCB types.

File Name: Supplementary Data 3

Description: The table lists 304 GSL structures identified by RPLC-MS/MS method at the chain composition level in porcine brain polar lipids. The data includes the m/z of [M + H]+, R.T., characteristic ions for chain composition identification, and mass errors.

File Name: Supplementary Data 4

Description: The table lists 157 GSL structures profiled by PB-MS/MS method at C=C and 2OH location level and 68 monounsaturated GSLs encompassing sphingosines in porcine brain polar lipids. It includes the m/z of [PBM + H]+, R.T. diagnostic ions for C=C and 2OH location, and %relative compositions of C=C location isomers in monounsaturated N-acyl chains.

File Name: Supplementary Data 5

Description: The table lists 277 GSL structures identified by RPLC-MS/MS method at the chain composition level in pooled human brain sample. The data includes the m/z of [M + H]+, R.T., characteristic ions for chain composition identification, and mass errors.

File Name: Supplementary Data 6

Description: The table includes 156 GSL structures profiled by PB-MS/MS method at C=C & 2OH location level and 80 monounsaturated GSLs encompassing sphingosines in pooled human brain sample. It includes the m/z of [PBM + H]+, R.T.s, diagnostic ions for C=C &2OH location, and %relative compositions of C=C location isomers in monounsaturated N-acyl chains.

File Name: Supplementary Data 7

Description: The table lists all the windows employed in each SWATH method and collision energy for each MS/MS experiment.
