# Supplementary material for: Illuminating the dark space of neutral glycosphingolipidome by selective enrichment and profiling at multi-structural levels
Source: Nat Commun. 2024 Jul 4;15:5627. doi: 10.1038/s41467-024-50014-8 (PMC11224418; doi:10.1038/s41467-024-50014-8)
Supplement: Supplementary file 11 — Reporting Summary [file 41467_2024_50014_MOESM11_ESM.pdf]

nature portfolio

Corresponding author(s): Yu Xia

Last updated by author(s): May 25,2024

Reporting Summary

Nature Portfolio wishes to improve the reproducibility of the work that we publish. This form provides structure for consistency and transparency in reporting. For further information on Nature Portfolio policies, see our Editorial Policies and the Editorial Policy Checklist.

Statistics

For all statistical analyses, confirm that the following items are present in the figure legend, table legend, main text, or Methods section.

n/a	Confirmed	
☐	☒	The exact sample size (n) for each experimental group/condition, given as a discrete number and unit of measurement
☐	☒	A statement on whether measurements were taken from distinct samples or whether the same sample was measured repeatedly
☐	☒	The statistical test(s) used AND whether they are one- or two-sided<br>*Only common tests should be described solely by name; describe more complex techniques in the Methods section.*
☒	☐	A description of all covariates tested
☒	☐	A description of any assumptions or corrections, such as tests of normality and adjustment for multiple comparisons
☐	☒	A full description of the statistical parameters including central tendency (e.g. means) or other basic estimates (e.g. regression coefficient) AND variation (e.g. standard deviation) or associated estimates of uncertainty (e.g. confidence intervals)
☐	☒	For null hypothesis testing, the test statistic (e.g. F, t, r) with confidence intervals, effect sizes, degrees of freedom and P value noted<br>*Give P values as exact values whenever suitable.*
☒	☐	For Bayesian analysis, information on the choice of priors and Markov chain Monte Carlo settings
☐	☒	For hierarchical and complex designs, identification of the appropriate level for tests and full reporting of outcomes
☒	☐	Estimates of effect sizes (e.g. Cohen's d, Pearson's r), indicating how they were calculated

Our web collection on statistics for biologists contains articles on many of the points above.

Software and code

Policy information about availability of computer code

Data collection	MS raw data were recorded with SCIEX OS 3.0.0.3339
Data analysis	SCIEX OS (3.0.0.3339) software was used for data analysis

For manuscripts utilizing custom algorithms or software that are central to the research but not yet described in published literature, software must be made available to editors and reviewers. We strongly encourage code deposition in a community repository (e.g. GitHub). See the Nature Portfolio guidelines for submitting code & software for further information.

Data

Policy information about availability of data

All manuscripts must include a data availability statement. This statement should provide the following information, where applicable:

- Accession codes, unique identifiers, or web links for publicly available datasets
- A description of any restrictions on data availability
- For clinical datasets or third party data, please ensure that the statement adheres to our policy

All data necessary to support the conclusions are available in the manuscript or supplementary information. The raw MS data are available from Figshare (https://doi.org/10.6084/m9.figshare.24771954). The derived MS data generated in this study have also been deposited in the MetaboLights database [www.ebi.ac.uk/metabolights/MTBLS10400]. Source data are provided with this paper.

Research involving human participants, their data, or biological material

Policy information about studies with human participants or human data. See also policy information about sex, gender (identity/presentation), and sexual orientation and race, ethnicity and racism.

Reporting on sex and gender	Thirteen female and fifteen males were included in the study. Due to the small sample size within each phenotype group, we were unable to conduct analyses based on sex and gender.
Reporting on race, ethnicity, or other socially relevant groupings	Race, ethnicity or other socially relevant groupings were not included as they have been suspected to have an association with brain glioma.
Population characteristics	The human brain tissue samples were obtained from a cohort of twenty-eight Asian patients, consisting of thirteen females and fifteen males. The age range of the participants was 32 to 80 years.
Recruitment	Not relevant. Sample were obtained from specimen bank of the hospital.
Ethics oversight	All patients recruited from Huashan Hospital, Fudan University, China, provided informed consent. The study protocol was approved by the Ethical Review Board of Tsinghua University (IRB No. 20180030).

Note that full information on the approval of the study protocol must also be provided in the manuscript.

Field-specific reporting

Please select the one below that is the best fit for your research. If you are not sure, read the appropriate sections before making your selection.

☒ Life sciences ☐ Behavioural & social sciences ☐ Ecological, evolutionary & environmental sciences

For a reference copy of the document with all sections, see nature.com/documents/nr-reporting-summary-flat.pdf

Life sciences study design

All studies must disclose on these points even when the disclosure is negative.

Sample size	These samples were solely used to illustrate the application of the developed lipidomic approach. A sample size of 22 was chosen for the mass spectrometry experiments to derive meaningful conclusions from the data, and this sample size also demonstrated the reproducibility of the lipid analysis results. A sample size of 6 was chosen for the western blot and qRT-PCR analysis to derive meaningful conclusions from the data.
Data exclusions	No data were excluded.
Replication	To ensure the reliability and robustness of our experimental results, we conducted analyses on 22 biological replicates for lipidomic analysis and 6 biological replicates for protein and gene expression analysis. All attempts at replication were successful.
Randomization	Glioma samples were categorized into IDH-mutant and IDH-wildtype samples based on the presence of the IDH mutation (R132H), diagnosed through IHC staining and PCR sequencing. Normal brain tissue samples are procured from the cancer-adjacent tissue excised during brain tumor surgery.
Blinding	The investigators were not blinded to group allocation during data collection and/or analysis. The group allocation is used to observe the regulation of glycosphingolipids in glioma phenotypes by selective enrichment method.

Reporting for specific materials, systems and methods

We require information from authors about some types of materials, experimental systems and methods used in many studies. Here, indicate whether each material, system or method listed is relevant to your study. If you are not sure if a list item applies to your research, read the appropriate section before selecting a response.

Materials & experimental systems

n/a	Involved in the study	
☐	☒	Antibodies
☒	☐	Eukaryotic cell lines
☒	☐	Palaeontology and archaeology
☒	☐	Animals and other organisms
☒	☐	Clinical data
☒	☐	Dual use research of concern
☒	☐	Plants

Methods

n/a	Involved in the study	
☒	☐	ChIP-seq
☒	☐	Flow cytometry
☒	☐	MRI-based neuroimaging

Antibodies

Antibodies used	FA2H polyclonal antibody 1:500 dilution (Proteintech, Catalog No. 1542-1-AP); CerS2 Antibody 1:1,000 dilution (BETHYL, Catalog No.A303-193A-T); Anti-SCD1 1:1,000 dilution (Abcam, Catalog No. ab236868, clone EPR21963)
Validation	Antibodies were validated via Western blot, per manufacturer recommendations, as follows: cell lysate from HeLa, HEK293T and Jurkat cells for CerS2; HeLa cells for SCD1; human brain and various lysates for FA2H.

Plants

Seed stocks	*Report on the source of all seed stocks or other plant material used. If applicable, state the seed stock centre and catalogue number. If plant specimens were collected from the field, describe the collection location, date and sampling procedures.*
Novel plant genotypes	*Describe the methods by which all novel plant genotypes were produced. This includes those generated by transgenic approaches, gene editing, chemical/radiation-based mutagenesis and hybridization. For transgenic lines, describe the transformation method, the number of independent lines analyzed and the generation upon which experiments were performed. For gene-edited lines, describe the editor used, the endogenous sequence targeted for editing, the targeting guide RNA sequence (if applicable) and how the editor was applied.*
Authentication	*Describe any authentication procedures for each seed stock used or novel genotype generated. Describe any experiments used to assess the effect of a mutation and, where applicable, how potential secondary effects (e.g. second site T-DNA insertions, mosiacism, off-target gene editing) were examined.*
